# Camelid Single-Domain Antibodies: Historical Perspective and Future Outlook

**DOI:** 10.3389/fimmu.2017.01589

**Published:** 2017-11-20

**Authors:** Mehdi Arbabi-Ghahroudi

**Affiliations:** ^1^Human Health Therapeutics Research Centre, National Research Council Canada, Ottawa, ON, Canada; ^2^Department of Biology, Carleton University, Ottawa, ON, Canada

**Keywords:** camelid single-domain antibody, heavy chain antibody, V_H_H, nanobody, antibody engineering, therapeutic antibody

## Abstract

Tremendous effort has been expended over the past two and a half decades to understand many aspects of camelid heavy chain antibodies, from their biology, evolution, and immunogenetics to their potential applications in various fields of research and medicine. In this article, I present a historical perspective on the development of camelid single-domain antibodies (sdAbs or V_H_Hs, also widely known as nanobodies) since their discovery and discuss the advantages and disadvantages of these unique molecules in various areas of research, industry, and medicine. Commercialization of camelid sdAbs exploded in 2001 with a flurry of patents issued to the Vrije Universiteit Brussel (VUB) and later taken on by the Vlaams Interuniversitair Instituut voor Biotechnologie (VIB) and, after 2002, the VIB-founded spin-off company, Ablynx. While entrepreneurial spirit has certainly catalyzed the exploration of nanobodies as marketable products, IP restrictions may be partially responsible for the relatively long time span between the discovery of these biomolecules and their entry into the pharmaceutical market. It is now anticipated that the first V_H_H-based antibody drug, Caplacizumab, a bivalent anti-vWF antibody for treating rare blood clotting disorders, may be approved and commercialized in 2018 or shortly thereafter. This elusive first approval, along with the expiry of key patents, may substantially alter the scientific and biomedical landscape surrounding camelid sdAbs and pave the way for their emergence as mainstream biotherapeutics.

## Introduction

The canonical view of antibodies as molecules composed of two heavy chains and two light chains was forever changed one day in 1989 following analysis of total and fractionated immunoglobulin G (IgG) molecules in the serum of a dromedary camel in the laboratory of Professor Raymond Hamers at the Vrije Universiteit Brussel (VUB). The serendipitous discovery of antibodies lacking a light chain [heavy chain-only antibodies (HCAbs)] occurred as part of a student-run project aimed at developing a serodiagnostic test for trypanosome infection in camels and water buffalos. The preliminary data showed that besides conventional IgG1 (MW ~150 kDa), two other immunoglobulin fractions (thereafter called IgG2 and IgG3; MW ~90 kDa) were present which contributed up to 75% of all serum IgGs (1–3). Comparative studies on the sera of new world camelids (*Lama glama* and *Lama pacos*) subsequently confirmed the presence of HCAbs, albeit at concentrations between 30 and 50% (1, 4–8). Following these exciting findings, it became essential to analyze the antigen-binding properties of these IgG fractions since the presence of truncated forms of heavy chain antibodies with no light chains, classically described as “heavy chain disease,” had been reported in human patients (9, 10). No functional activity was reported for the pathogenic heavy chain antibodies in these patients, as these proteins were shown to bear extensive internal deletions in the variable (VH) and the first constant region (CH1) domains. By contrast, antibodies from camelids exposed to *Trypanosoma evansi* demonstrated strong binding activity in the IgG2 and IgG3 heavy chain-only fractions as shown by radio-immunoprecipitation and blotting experiments (1).

In two subsequent reports, phage-display technology and high-resolution crystallography were utilized to (a) build a phage-display library from the lymphocytes of immunized camels and isolate monomeric antigen-specific V_H_H domains in the absence of the constant regions (11) and (b) solve crystal structures of an unliganded V_H_H (12) and a V_H_H:lysozyme complex, reported simultaneously by the VUB team and a Dutch–French research group (13). The term V_H_H was originally introduced by the VUB team in 1994 to indicate a VH domain derived from camelid heavy chain antibodies. The feasibility of isolating stable and soluble V_H_H domains with nanomolar affinities against lysozyme and tetanus toxoid showed very early on the promise of these molecules as high-affinity binding moieties. Crystallography studies revealed additional salient features of an anti-lysozyme V_H_H, including deep penetration of its long third complementarity-determining region (CDR3) into the active site of the enzyme; this feature had rarely been seen with conventional antibodies and required a fundamental deviation from known human canonical CDR1 structure (13). Further evidence of the unique antigen recognition behavior of V_H_H domains (including enzyme inhibition) was published over the next several years (11, 14, 15), suggesting that V_H_Hs might probe different sets of epitopes on proteins compared with conventional antibodies. Key proof of concept for producing bivalent/bispecific V_H_H modalities *via* genetic fusion (using camelid short and long hinge sequences) of anti-lyzozyme and/or anti-tetanus toxin V_H_Hs was also established very early on (14).

## Molecular Ontogeny of Camelid HCAbs

Molecular biology techniques were subsequently applied to decipher the DNA sequences of HCAbs. The sequencing results showed that nature had designed HCAbs as an additional arm of the immune systems of camelid ungulates over the course of their evolutionary history. The consensus of these studies suggested camelid HCAbs possessed: (a) no CH1 domain, and therefore, a direct connection of the rearranged V_H_H exon to the hinge region; (b) one of two types of long (IgG2) and short (IgG3) hinge isotypes; (c) specific conserved amino acid substitutions in framework region 2 (FR2), mainly at VH positions that make contact with the VL in classical antibodies, including Kabat positions 37, 44, 45, and 47; and (d) potentially different CDR3 amino acid composition and a broader length distribution for CDR3 compared to the heavy chains of conventional antibodies (1, 16, 17).

Later genomic studies shed light on the origin of HCAbs in dromedary camels and alpacas. It is now established that HCAbs are produced from the same *igh* locus as conventional antibodies but with distinct sets of genes for the generation of HCAbs. It is estimated that alpaca and dromedary genomes contain ~17 and ~40 V_H_H genes, respectively, with an identical organization of the genes that produce conventional antibodies (18, 19). The CH1 exon is present in the genomic DNA of HCAbs but a point mutation (G to A) at the 5′ end of the CH1-hinge intron disrupts the consensus splicing site (GT) and causes omission of this region during splicing (3, 18, 20–22). A complete picture of camelid germline V gene repertoires of heavy and light chains and the classification of VH and V_H_H genes is still missing. Published genomic and cDNA data have so far shown that camelid V_H_H genes are highly homologous to the human VH3 family of clan III with the exception of several key amino acid substitutions in FR2, namely, Val37 → Phe/Tyr, Gly44 → Glu, Leu45 → Arg, and Trp47 → Gly (Kabat numbering), and are encoded by a distinct subset of germline V genes. Preliminary investigations of published llama V_H_H sequences classified them into four subfamilies by sequence similarity, and many of the earliest-described V_H_H features such as long CDR3s, additional disulfide bridges, and particular canonical structures of CDR1–3 were shown to be subfamily specific (17, 23). Subsequent studies in alpaca identified at least three V gene subgroups of the alpaca *igh* locus: IGHV1, IGHV2, and IGHV3 which are equivalent to the human IGHV families within clan I (VH families 2, 4, 6), II (VH families 1, 5, 7), and III (VH family 3), respectively, based on sequence homology. The alpaca V_H_H genes clustered into six subsets by sequence similarity, but all are homologous to human IGHV3 genes (18). Furthermore, recent investigations have demonstrated the presence of genes belonging to IGHV families 1, 3, and 4 (human clan I and III) in llama and alpaca, and in addition, uncovered new camelid V genes highly homologous to the human IGHV5 and IGHV7 families (human clan II); however, no genes similar to human families 2 or 6 (within human clan I) were found (24). Interestingly, a novel promiscuous class of V genes in camelids was identified that is closely related to the human VH4 family (clan I). These VH4 homologs contribute largely to the classical antibody repertoire and lack the hallmark solubilizing V_H_H residues in FR2. Nevertheless, antigen-specific VH4-family fragments with V_H_H-like stability and solubility were isolated from an immune llama library (25). In the absence of a complete set of camelid germline VH and V_H_H genes, most immunogenetic studies have relied on comparisons with human germline genes.

The consensus of immunogenetic studies of camelid HCAbs is that repertoire diversification of these molecules may involve (a) a large number of unique V_H_H gene segments recombining with DH and JH minigenes, possibly with additional non-templated nucleotide insertions leading to longer CDR3 loops; (b) somatic hypermutation, potentially of extended CDR1 regions compared with conventional antibodies; (c) acquisition of non-canonical cysteine residues in the CDRs and FR2; and (d) involvement of FR2 residues in antigen binding and in structuring the CDR3 loop (3, 22, 26, 27). In agreement with immunogenetic analyses, several structural studies have suggested that due to the loss of VL domains, V_H_H paratopes have acquired a higher structural complexity by involving more residues in antigen binding compared to classical VHs (27). As for the evolutionary origin of HCAbs, it is difficult to draw solid conclusions but several hypotheses have been proposed. A common theme among most of these has been the need for generating or expanding a new antigen-binding repertoire in *Camelidae* to address certain antigenic challenges, e.g., cryptic epitopes of commonly encountered pathogens. Phylogenetic analyses have confirmed that HCAbs diverged from conventional antibodies as a result of recent adaptive changes (22, 27–29).

## History of the Development of Camelid Single-Domain Antibodies (sdAbs) as Therapeutics

**Figure 1:**
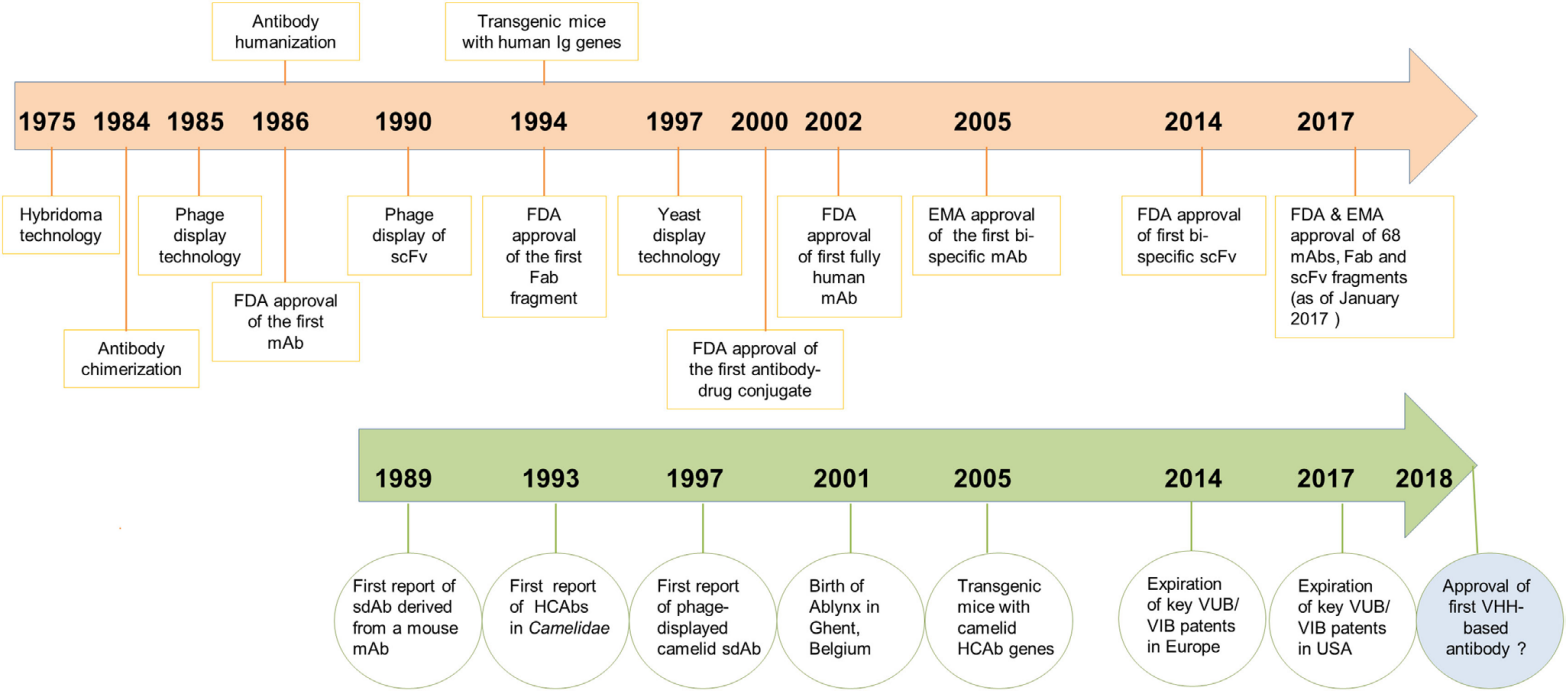
Chronological timeline of major scientific developments in the field of antibody engineering since the discovery of monoclonal antibodies (mAbs) in 1975 leading to the regulatory approval of mAbs, antigen-binding fragments (Fabs) and scFvs as therapeutics. Developments for mAbs are shown in orange and developments of V_H_Hs/heavy chain-only antibodies (HCAbs) in green. Regulatory approval of the first V_H_H-based antibody drug is expected in 2018.

Prior to the discovery of HCAbs, a single report describing the concept of sdAbs was published by Sally Ward and colleagues in 1989 (30), when they showed that VH domains from an immunized mouse, in the absence of a VL domain, could bind specifically to lysozyme and keyhole limpet hemocyanin. However, poor VH domain stability and solubility, as well as weak antigen-binding affinity compared to its fragment variable region counterpart (Fv) or to the parent antibody, were major impediments to any commercial applications (Figure 1).

From a historical perspective, development of camelid V_H_Hs as drugs has gone through three major phases. The first 10 years (1993–2003) can be classified as the exploratory phase, which coincided with the founding of Ablynx in December 2001 as a spin-off company from the Vlaams Interuniversitair Instituut voor Biotechnologie. The main developments in the first decade included: (i) the first description of V_H_Hs (1); (ii) sequence analyses of V_H_Hs with identification of V_H_H germline gene segments and classification of V_H_H gene subfamilies (16, 20, 23); (iii) adaptation of phage-display technology to V_H_Hs (11) and isolation of antigen-specific V_H_Hs, including several enzyme inhibitors (12, 15); (iv) solving the crystal structure of several V_H_H:antigen complexes (13, 31–34); (v) development of methods for expression of V_H_Hs in bacteria and yeast systems and for biophysical characterization of V_H_Hs (35, 36); and (vi) the use of V_H_Hs as reagents in immunoaffinity purification and immuno-perfusion (37).

During the second phase of development (2003–2013), V_H_Hs began to receive more attention and publications in this area grew dramatically, surpassing 1,000 by 2013 [Ref. (38) and personal investigation on Web of Science]. Interestingly, a large and diverse group of countries and institutions (close to 50) were responsible for research on camelid V_H_Hs during this time, mainly for the purpose of exploring their potential applications in research, biotechnology, and medicine (38). The major hallmark of this decade was the start of preclinical and clinical studies of several nanobodies by Ablynx and others as therapeutics and imaging reagents (39, 40), including V_H_Hs against (i) blood glycoprotein vWF to control platelet aggregation and clot formation; (ii) viral infection (RSV); (iii) venom toxins; (iv) IL6-R for treatment of rheumatoid arthritis; and (v) the use of radiolabeled nanobodies for Her2+ tumor imaging. There were major technological advancements made in the expression of V_H_Hs in heterologous systems and in creating an array of bi- and multivalent V_H_Hs with superior efficacy during this decade.

Now in the third phase of development (2014–present), publications continue to grow and more V_H_Hs have entered into clinical trials or advanced closer to the market. The main patent claims on camelid antibody fragments expired in the summer of 2014 in Europe and in the summer of 2017 in America. Ablynx has expanded its collaborations with large biophama players, such as Merck, Boehringer Ingelheim, Sanofi, and so on, with more than 20 preclinical and clinical programs. It is expected that the first V_H_H-based drug (Caplacizumab; bivalent anti-vWF nanobody for treating rare blood clotting disorders) will reach the market sometime in 2018 (www.ablynx.com). Meanwhile, IP limitations on the composition of matter of V_H_Hs are diminishing and more biotechnology companies (39) are showing interest in commercialization of these domain antibodies as therapeutics, diagnostics, and research reagents (Figure 1).

## Camelid sdAbs: Pros, Cons, and Applications

Immunization of *Camelidae* against targets of interest leads to the *in vivo* maturation of HCAb and conventional antibody repertoires. Construction of phage-display libraries is performed by cloning of amplified V_H_H repertoires with minimal modification, thus presenting an authentic picture of *in vivo*-matured heavy chain repertoire diversity. By contrast, in both scFv libraries (requiring the artificial joining of VH and VL domains by a synthetic linker) and antigen-binding fragment (Fab) libraries derived from conventional antibody repertoires, natural VH–VL pairings are usually lost. The potential for direct cloning of V_H_H repertoires from immunized camelids, the smaller library sizes required to capture the immune V_H_H repertoire, the stability of the libraries, the feasibility of displaying V_H_Hs on a phage or alternative display formats, and the ease of sub-cloning and expression of antigen-specific V_H_Hs are among the major technical advantages of the camelid V_H_H platform over conventional antibody platforms.

Key characteristics of V_H_Hs include their high affinity and specificity (equivalent to conventional antibodies), high thermostability, good solubility and strictly monomeric behavior, small size (2.5 nm in diameter and about 4 nm in length; ~15 kDa), relatively low production cost, ease of genetic engineering, format flexibility or modularity, low immunogenicity, and a higher penetration rate into tissues (3, 41–44). The short half-life of V_H_Hs in blood circulation is well suited to certain applications such as tumor imaging or delivery of toxin or radioisotopes to diseased tissues where rapid clearance is required. However, the pharmacokinetic behavior of V_H_Hs can also be improved by extending their half-lives using different formatting options, including PEGylation or fusion to serum albumin or an anti-serum albumin moiety (43, 45, 46). The immunogenicity of V_H_Hs domains can also be minimized by humanization (47–49). As with all antibodies of non-human species origin (and even fully human antibodies), immunogenicity and toxicity must be investigated empirically for humanized V_H_Hs. A complete picture of the immunogenicity of non-humanized and humanized camelid V_H_Hs is lacking due to insufficient data, but anti-drug immune responses may have been a major reason for the clinical failure of a humanized tetravalent Nanobody^®^targeting the DR5 receptor (50). As of 2016, V_H_Hs have been isolated against more than 120 therapeutically important targets relevant to oncology, *in vivo* imaging, hematology, infectious diseases, neurological, and inflammatory disorders, with some in advanced stages of clinical trials (39).

One of the unique characteristics of V_H_Hs is their ability to target antigenic epitopes at locations which are difficult to access by large molecules such as conventional monoclonal antibodies (mAbs). Examples include intracellular targets (51, 52) or epitopes concealed from mAbs in protein structures (53), G protein-coupled receptors (54, 55), and ion channels (3). V_H_Hs are ideally suited for such applications due to their small size, target specificity, and long CDR3 loops, bypassing many drawbacks related to small-molecule synthetic drugs such as fine specificity and off-target toxicity (56). As “intrabodies,” V_H_Hs are also ideally suited for cytosolic expression due to their ability to fold in the reducing intracellular environment. This feature likely reflects the single disulfide linkage present in the V_H_H domain, as compared to the multi-domain structure and multiple disulfide linkages of conventional antibodies, and may not be completely general to all V_H_Hs but appears to be quite common; intracellular expression of V_H_Hs has been widely and productively exploited for *in vivo* cellular imaging (5, 57) as well as to inhibit the function of viral proteins (58, 59). There have been several excellent reviews covering V_H_H applications in different areas of basic and applied research and a detailed description of each application is beyond the scope of this article (3, 39, 41, 43, 57, 60–65).

V_H_Hs are also well suited in the generation of bi- and multispecific antibodies. In the field of antibody therapeutics, it is now widely accepted that monotherapy of cancer and other diseases may not result in effective outcomes, in particular due to the problem of acquired resistance (66, 67). Bispecific antibodies provide a possible solution in which they could bind simultaneously to a tumor-associated antigen and another activating molecule, e.g., CD3 on T cells, leading to tumor killing/lysis through lymphocyte recruitment, or alternatively, could target two or more tumor epitopes (bi-paratopic) or antigens simultaneously. Bispecific V_H_Hs may be uniquely positioned for these applications given their simple design and small size relative to other antibody fragments, which may result in better solid tumor penetration rates, homogeneous production at high yield in microbial systems, and ease of fusion to a heterodimerization motif, therefore bypassing issues related to some linker peptides such as aggregation and immunogenicity (45, 66, 68, 69). Interestingly, all of the V_H_H-based therapeutic candidates in clinical trials are composed of bivalent, trivalent, or higher valency formats (39). It has been shown that some V_H_Hs, when properly selected, are able to transmigrate through human brain endothelial cell layers spontaneously and, possibly through a receptor-mediated process (70–72); bispecific molecules incorporating these V_H_Hs can, thus, deliver attached cargo (e.g., therapeutics) into the brain in rodents (73).

Despite the many advantages of V_H_Hs, there are several drawbacks to be considered as well. The fact that the antigen-binding paratope of camelid HCAbs has been restricted to a single domain of about 110 amino acids will automatically put more weight on each and every residue in the V_H_H domain. The extended CDR1, longer CDR3, involvement of FR2 in antigen binding and shaping the CDR3 loop, the role of the “CDR4” (residues 76–80) loop in antigen binding, and extensive somatic hypermutation are some of the evolutionary mechanisms adapted to compensate repertoire diversity due to the lack of a VL domain (3). Therefore, there may be limitations on the extent of manipulation and engineering that can be tolerated by antigen-specific V_H_Hs. For example, complete humanization of camelid V_H_Hs involving the mutation of residues outside the antigen-binding loops often drastically compromises antigen-binding affinity, V_H_H stability, and the expression yield (unpublished data). A survey of the literature clearly demonstrates that almost all V_H_Hs isolated to date have originated from direct camelid immunization, or from large naïve camelid libraries, although recently, successful isolation of V_H_Hs from synthetic or semi-synthetic libraries against a number of protein antigens has also been reported (74–77). All available pieces of evidence support the notion that the V_H_H domain is a highly complex molecule and that each amino acid (depending on its position) may have direct and indirect effects on the molecule’s stability and structural integrity, as well as on antigen-binding affinity and specificity.

Another limitation of V_H_Hs is their low propensity to bind small molecules, likely due to their dominant convex surface topology as compared to the flat or concave topologies found on conventional antibody fragments (e.g., scFv, Fab). In a number of llama immunization trials, we and others have been able to generate strong conventional immune responses, but rather weak HCAb responses, against several haptens and carbohydrate antigens (unpublished data). However, repeated immunization of camelids with small molecules conjugated or fused to larger proteins has led to the successful isolation of V_H_Hs against caffeine (78), red dye (79), and linear peptides (80, 81) with affinities ranging from micromolar to low nanomolar. The biophysicochemical properties of V_H_Hs suggest that they would be well suited to many immunodiagnostic platforms for detecting small molecules and environmental chemicals; however, isolation of high-affinity V_H_Hs suitable for such applications seems to be a difficult task, although not impossible (3, 64, 65, 78, 82, 83). Immunization of large animals and heterogeneity in immune responses among individual outbred animals is another consideration which is important when alternative immunization techniques such as DNA immunization are applied. DNA immunization has had limited success in camelid and other large animals and reproducibility is often a major issue to be tackled (84–87). To overcome this limitation, transgenic mice bearing either a rearranged dromedary γ2a chain or hybrid llama/human antibody loci have been generated that produce a form of dromedary or human heavy chain antibodies (88–90).

## Camelid sdAbs Versus mAbs

The first therapeutic mAb, Orthoclone OKT3, a murine IgG2a for the prevention of kidney transplant rejection, hit the market little more than a decade after the discovery of hydridoma technology in 1975 (91–94). Currently, mAbs constitute about half of marketed biological products and, as of January 2017, 68 mAbs have been approved by the Food and Drug Administration (FDA) in the USA and/or by the European Medicine Agency (EMA) in Europe. The projected global sales of mAbs will be close to $100 billion in 2017 (44, 95). The lack of restrictive IP on the original technology is considered by many as a driving force that allowed researchers to develop effective research tools and diagnostic mAb-based reagents without limitation. The introduction of antibody fragments, such as Fab and scFv (the “second generation” of antibodies), combined with the power of phage-display technology in the late 1990s, opened new horizons in the world of antibodies and empowered researchers with the ability to clone the entire immunoglobulin repertoire of mammalian immune B cells and to isolate specific antibody fragments virtually against any target (96–98). This technology led to the development of the first FDA-approved fully human mAb, Humira, which was obtained from a phage-displayed human antibody library 12 years after the initial paper by McCafferty and co-workers on the construction of phage-displayed human antibody libraries (99–101). Further developments in antibody engineering have so far resulted in three FDA-approved therapeutic Fabs (95).

Overwhelming evidence in the literature suggests that camelid V_H_Hs, as the so-called “third generation” of antibodies, have many added features that supersede those of conventional mAbs and antibody fragments (Fab and scFv). Although V_H_Hs have already been commercialized for non-medical applications (63, 102), the research and medical communities eagerly await the first V_H_H-based therapeutic to gain approval. If we consider the 9- to 13-year time span between the discovery of the key technology enabling conventional mAbs (hybridoma technology) and the FDA-approval of a mAb or an antibody fragment, a longer time has been required for the development of the first V_H_H-based therapeutic. It is unclear if technical challenges, regulatory hurdles, or the need to define a unique niche/indication for V_H_Hs, have been involved in the prolonged delay of the first V_H_H-based therapeutic. It is obvious that issues related to downstream processing, stability, immunogenicity, toxicity, safety, and potency of a V_H_H-based therapeutic product will be doubly scrutinized by FDA and EMA since it would represent the first product of its kind to enter the market. The fact that the first potential Ablynx product is an engineered bivalent anti-vWF nanobody and is produced in a microbial system may have raised additional red flags for the approving regulatory bodies.

## Concluding Remarks

Over a quarter century has passed since the first observation by Hamers and colleagues of camelid HCAbs. This finding was a significant milestone in the field of antibody engineering and opened many new opportunities and applications. It was also instrumental in reviving the concept of sdAbs, which had been originally suggested by Ward et al. a few years earlier. The unique and extraordinary features of HCAbs and their antigen-binding domains (V_H_Hs) have with no doubt attracted many researchers and commercial entities to the field of antibody engineering. V_H_Hs are now closer than ever to approval as pharmaceutical drugs to fight a wide range of diseases, including cancer, inflammation, hematology, and respiratory diseases, with five V_H_H-based drugs in various stages of clinical development. V_H_Hs have also been shown to be effective as therapeutics against infectious disease, particularly in viral therapy, as well as robust reagents in the field of diagnostic and imaging applications. While the commercial applications of V_H_Hs have been slowed by IP limitations, it is probable that demand, as well as extensive research on these antibody domains, will ultimately supersede these limitations and bring many more of these molecules into use as biopharmaceutical reagents within the next decade.

## Author Contributions

MA-G conceived and wrote the manuscript.

## Conflict of Interest Statement

The author declares that the research was conducted in the absence of any commercial or financial relationships that could be construed as a potential conflict of interest.

## References

[1] Hamers-Casterman C Atarhouch T Muyldermans S Robinson G Hamers C Songa EB Naturally occurring antibodies devoid of light chains. Nature (1993) 363:446–8. 10.1038/363446a0 8502296

[2] Wernery U Camelid immunoglobulins and their importance for the new-born – a review. J Vet Med B Infect Dis Vet Public Health (2001) 48:561–8. 10.1111/j.1439-0450.2001.00478.x 11708675

[3] Muyldermans S. Nanobodies: natural single-domain antibodies. Annu Rev Biochem (2013) 82:775–97. 10.1146/annurev-biochem-063011-092449 23495938

[4] van der Linden R de Geus B Stok W Bos W van Wassenaar D Verrips T Induction of immune responses and molecular cloning of the heavy chain antibody repertoire of *Lama glama*. J Immunol Methods (2000) 240:185–95. 10.1016/S0022-1759(00)00188-5 10854612

[5] Rothbauer U Zolghadr K Tillib S Nowak D Schermelleh L Gahl A Targeting and tracing antigens in live cells with fluorescent nanobodies. Nat Methods (2006) 3:887–9. 10.1038/nmeth953 17060912

[6] Maass DR Sepulveda J Pernthaner A Shoemaker CB. Alpaca (*Lama pacos*) as a convenient source of recombinant camelid heavy chain antibodies (V_H_Hs). J Immunol Methods (2007) 324:13–25. 10.1016/j.jim.2007.04.008 17568607 PMC2014515

[7] De Simone EA Saccodossi N Ferrari A Leoni J. Development of ELISAs for the measurement of IgM and IgG subclasses in sera from llamas (*Lama glama*) and assessment of the humoral immune response against different antigens. Vet Immunol Immunopathol (2008) 126:64–73. 10.1016/j.vetimm.2008.06.015 18692907

[8] Blanc MR Anouassi A Ahmed Abed M Tsikis G Canepa S Labas V A one-step exclusion-binding procedure for the purification of functional heavy-chain and mammalian-type gamma-globulins from camelid sera. Biotechnol Appl Biochem (2009) 54:207–12. 10.1042/BA20090208 19824883

[9] Franklin EC Lowenstein J Bigelow B Meltzer M Heavy chain disease – a new disorder of serum gamma-globulins: report of the first case. Am J Med (1964) 37:332–50. 10.1016/0002-9343(64)90191-3 14209281

[10] Alexander A Steinmetz M Barritault D Frangione B Franklin EC Hood L gamma heavy chain disease in man: cDNA sequence supports partial gene deletion model. Proc Natl Acad Sci U S A (1982) 79:3260–4. 10.1073/pnas.79.10.3260 6808505 PMC346395

[11] Arbabi Ghahroudi M Desmyter A Wyns L Hamers R Muyldermans S. Selection and identification of single domain antibody fragments from camel heavy-chain antibodies. FEBS Lett (1997) 414:521–6. 10.1016/S0014-5793(97)01062-4 9323027

[12] Spinelli S Frenken L Bourgeois D de Ron L Bos W Verrips T The crystal structure of a llama heavy chain variable domain. Nat Struct Biol (1996) 3:752–7. 10.1038/nsb0996-752 8784347

[13] Desmyter A Transue TR Ghahroudi MA Thi MH Poortmans F Hamers R Crystal structure of a camel single-domain VH antibody fragment in complex with lysozyme. Nat Struct Biol (1996) 3:803–11. 10.1038/nsb0996-803 8784355

[14] Arbabi Ghahroudi M Generation and Characterization of Phage-Displayed Camel Single-Domain Antibodies [Ph.D. Dissertation]. Brussels (Belgium): Vrije Universiteit Brussel (VUB) (1996).

[15] Lauwereys M Arbabi Ghahroudi M Desmyter A Kinne J Holzer W De Genst E Potent enzyme inhibitors derived from dromedary heavy-chain antibodies. EMBO J (1998) 17:3512–20. 10.1093/emboj/17.13.3512 9649422 PMC1170688

[16] Muyldermans S Atarhouch T Saldanha J Barbosa JA Hamers R. Sequence and structure of VH domain from naturally occurring camel heavy chain immunoglobulins lacking light chains. Protein Eng (1994) 7:1129–35. 10.1093/protein/7.9.1129 7831284

[17] Vu KB Ghahroudi MA Wyns L Muyldermans S. Comparison of llama VH sequences from conventional and heavy chain antibodies. Mol Immunol (1997) 34:1121–31. 10.1016/S0161-5890(97)00146-6 9566760

[18] Achour I Cavelier P Tichit M Bouchier C Lafaye P Rougeon F. Tetrameric and homodimeric camelid IgGs originate from the same IgH locus. J Immunol (2008) 181:2001–9. 10.4049/jimmunol.181.3.2001 18641337

[19] Nguyen VK Hamers R Wyns L Muyldermans S Camel heavy-chain antibodies: diverse germline V_H_H and specific mechanisms enlarge the antigen-binding repertoire. EMBO J (2000) 19:921–30. 10.1093/emboj/19.5.921 10698934 PMC305632

[20] Nguyen VK Muyldermans S Hamers R. The specific variable domain of camel heavy-chain antibodies is encoded in the germline. J Mol Biol (1998) 275:413–8. 10.1006/jmbi.1997.1477 9466919

[21] De Genst E Saerens D Muyldermans S Conrath K. Antibody repertoire development in camelids. Dev Comp Immunol (2006) 30:187–98. 10.1016/j.dci.2005.06.010 16051357

[22] Conrath KE Wernery U Muyldermans S Nguyen VK. Emergence and evolution of functional heavy-chain antibodies in *Camelidae*. Dev Comp Immunol (2003) 27:87–103. 10.1016/S0145-305X(02)00071-X 12543123

[23] Harmsen MM Ruuls RC Nijman IJ Niewold TA Frenken LG de Geus B. Llama heavy-chain V regions consist of at least four distinct subfamilies revealing novel sequence features. Mol Immunol (2000) 37:579–90. 10.1016/S0161-5890(00)00081-X 11163394

[24] Klarenbeek A El Mazouari K Desmyter A Blanchetot C Hultberg A de Jonge N Camelid Ig V genes reveal significant human homology not seen in therapeutic target genes, providing for a powerful therapeutic antibody platform. MAbs (2015) 7:693–706. 10.1080/19420862.2015.1046648 26018625 PMC4622956

[25] Deschacht N De Groeve K Vincke C Raes G De Baetselier P Muyldermans S. A novel promiscuous class of camelid single-domain antibody contributes to the antigen-binding repertoire. J Immunol (2010) 184:5696–704. 10.4049/jimmunol.0903722 20404276

[26] Muyldermans S. Single domain camel antibodies: current status. J Biotechnol (2001) 74:277–302. 11526908 10.1016/s1389-0352(01)00021-6

[27] Nguyen VK Su C Muyldermans S van der Loo W. Heavy-chain antibodies in *Camelidae*; a case of evolutionary innovation. Immunogenetics (2002) 54:39–47. 10.1007/s00251-002-0433-0 11976790

[28] Daley LP Gagliardo LF Duffy MS Smith MC Appleton JA. Application of monoclonal antibodies in functional and comparative investigations of heavy-chain immunoglobulins in new world camelids. Clin Diagn Lab Immunol (2005) 12:380–6. 15753251 10.1128/CDLI.12.3.380-386.2005 PMC1065209

[29] Flajnik MF Deschacht N Muyldermans S A case of convergence: why did a simple alternative to canonical antibodies arise in sharks and camels? PLoS Biol (2011) 9:e1001120 10.1371/journal.pbio.1001120 21829328 PMC3149040

[30] Ward ES Gussow D Griffiths AD Jones PT Winter G. Binding activities of a repertoire of single immunoglobulin variable domains secreted from *Escherichia coli*. Nature (1989) 341:544–6. 10.1038/341544a0 2677748

[31] Spinelli S Desmyter A Frenken L Verrips T Tegoni M Cambillau C. Domain swapping of a llama V_H_H domain builds a crystal-wide beta-sheet structure. FEBS Lett (2004) 564:35–40. 10.1016/S0014-5793(04)00304-7 15094039

[32] Decanniere K Desmyter A Lauwereys M Ghahroudi MA Muyldermans S Wyns L. A single-domain antibody fragment in complex with RNase A: non-canonical loop structures and nanomolar affinity using two CDR loops. Structure (1999) 7:361–70. 10.1016/S0969-2126(99)80049-5 10196124

[33] Desmyter A Spinelli S Payan F Lauwereys M Wyns L Muyldermans S Three camelid V_H_H domains in complex with porcine pancreatic α-amylase: inhibition and versatility of binding topology. J Biol Chem (2002) 277:23645–50. 10.1074/jbc.M202327200 11960990

[34] Desmyter A Decanniere K Muyldermans S Wyns L. Antigen specificity and high affinity binding provided by one single loop of a camel single-domain antibody. J Biol Chem (2001) 276:26285–90. 10.1074/jbc.M102107200 11342547

[35] Perez JM Renisio JG Prompers JJ van Platerink CJ Cambillau C Darbon H Thermal unfolding of a llama antibody fragment: a two-state reversible process. Biochemistry (2001) 40:74–83. 10.1021/bi0009082 11141058

[36] Dumoulin M Conrath K Van Meirhaeghe A Meersman F Heremans K Frenken LG Single-domain antibody fragments with high conformational stability. Protein Sci (2002) 11:500–15. 10.1110/ps.34602 11847273 PMC2373476

[37] Verheesen P ten Haaft MR Lindner N Verrips CT de Haard JJ. Beneficial properties of single-domain antibody fragments for application in immunoaffinity purification and immuno-perfusion chromatography. Biochim Biophys Acta (2003) 1624:21–8. 10.1016/j.bbagen.2003.09.006 14642809

[38] Eyer L Hruska K Single-domain antibody fragments derived from heavy-chain antibodies: a review. Vet Med (2012) 9:439–513.

[39] Steeland S Vandenbroucke RE Libert C. Nanobodies as therapeutics: big opportunities for small antibodies. Drug Discov Today (2016) 21:1076–113. 10.1016/j.drudis.2016.04.003 27080147

[40] D’Huyvetter M Aerts A Xavier C Vaneycken I Devoogdt N Gijs M Development of ^177^Lu-nanobodies for radioimmunotherapy of HER2-positive breast cancer: evaluation of different bifunctional chelators. Contrast Media Mol Imaging (2012) 7:254–64. 10.1002/cmmi.491 22434639

[41] Wesolowski J Alzogaray V Reyelt J Unger M Juarez K Urrutia M Single domain antibodies: promising experimental and therapeutic tools in infection and immunity. Med Microbiol Immunol (2009) 198:157–74. 10.1007/s00430-009-0116-7 19529959 PMC2714450

[42] Saerens D Ghassabeh GH Muyldermans S. Single-domain antibodies as building blocks for novel therapeutics. Curr Opin Pharmacol (2008) 8:600–8. 10.1016/j.coph.2008.07.006 18691671

[43] Chakravarty R Goel S Cai W Nanobody: the “magic bullet” for molecular imaging? Theranostics (2014) 4:386–98. 10.7150/thno.8006 24578722 PMC3936291

[44] Fernandes CFC Pereira SDS Luiz MB Zuliani JP Furtado GP Stabeli RG. Camelid single-domain antibodies as an alternative to overcome challenges related to the prevention, detection, and control of neglected tropical diseases. Front Immunol (2017) 8:653. 10.3389/fimmu.2017.00653 28649245 PMC5465246

[45] Holt LJ Herring C Jespers LS Woolven BP Tomlinson IM. Domain antibodies: proteins for therapy. Trends Biotechnol (2003) 21:484–90. 10.1016/j.tibtech.2003.08.007 14573361

[46] Harmsen MM van Solt CB Fijten HP van Keulen L Rosalia RA Weerdmeester K Passive immunization of guinea pigs with llama single-domain antibody fragments against foot-and-mouth disease. Vet Microbiol (2007) 120:193–206. 10.1016/j.vetmic.2006.10.029 17127019

[47] Vaneycken I D’Huyvetter M Hernot S De Vos J Xavier C Devoogdt N Immuno-imaging using nanobodies. Curr Opin Biotechnol (2011) 22:877–81. 10.1016/j.copbio.2011.06.009 21726996

[48] Hassanzadeh-Ghassabeh G Devoogdt N De Pauw P Vincke C Muyldermans S Nanobodies and their potential applications. Nanomedicine (Lond) (2013) 8:1013–26. 10.2217/nnm.13.86 23730699

[49] Vincke C Loris R Saerens D Martinez-Rodriguez S Muyldermans S Conrath K. General strategy to humanize a camelid single-domain antibody and identification of a universal humanized nanobody scaffold. J Biol Chem (2009) 284:3273–84. 10.1074/jbc.M806889200 19010777

[50] Papadopoulos KP Isaacs R Bilic S Kentsch K Huet HA Hofmann M Unexpected hepatotoxicity in a phase I study of TAS266, a novel tetravalent agonistic Nanobody^(R)^ targeting the DR5 receptor. Cancer Chemother Pharmacol (2015) 75:887–95. 10.1007/s00280-015-2712-0 25721064

[51] McGonigal K Tanha J Palazov E Li S Gueorguieva-Owens D Pandey S. Isolation and functional characterization of single domain antibody modulators of caspase-3 and apoptosis. Appl Biochem Biotechnol (2009) 157:226–36. 10.1007/s12010-008-8266-4 18553063

[52] Staus DP Wingler LM Strachan RT Rasmussen SG Pardon E Ahn S Regulation of β2-adrenergic receptor function by conformationally selective single-domain intrabodies. Mol Pharmacol (2014) 85:472–81. 10.1124/mol.113.089516 24319111 PMC3935154

[53] Stijlemans B Conrath K Cortez-Retamozo V Van Xong H Wyns L Senter P Efficient targeting of conserved cryptic epitopes of infectious agents by single domain antibodies: African trypanosomes as paradigm. J Biol Chem (2004) 279:1256–61. 10.1074/jbc.M307341200 14527957

[54] Bradley ME Dombrecht B Manini J Willis J Vlerick D De Taeye S Potent and efficacious inhibition of CXCR2 signaling by biparatopic nanobodies combining two distinct modes of action. Mol Pharmacol (2015) 87:251–62. 10.1124/mol.114.094821 25468882

[55] Manglik A Kobilka BK Steyaert J. Nanobodies to study G protein-coupled receptor structure and function. Annu Rev Pharmacol Toxicol (2017) 57:19–37. 10.1146/annurev-pharmtox-010716-104710 27959623 PMC5500200

[56] Baker M. Upping the ante on antibodies. Nat Biotechnol (2005) 23:1065–72. 10.1038/nbt0905-1065 16151393

[57] Beghein E Gettemans J. Nanobody technology: a versatile toolkit for microscopic imaging, protein-protein interaction analysis, and protein function exploration. Front Immunol (2017) 8:771. 10.3389/fimmu.2017.00771 28725224 PMC5495861

[58] Rossey I Gilman MS Kabeche SC Sedeyn K Wrapp D Kanekiyo M Potent single-domain antibodies that arrest respiratory syncytial virus fusion protein in its prefusion state. Nat Commun (2017) 8:14158. 10.1038/ncomms14158 28194013 PMC5316805

[59] Darling TL Sherwood LJ Hayhurst A. Intracellular crosslinking of filoviral nucleoproteins with Xintrabodies restricts viral packaging. Front Immunol (2017) 8:1197. 10.3389/fimmu.2017.01197 29021793 PMC5623874

[60] Holliger P Hudson PJ. Engineered antibody fragments and the rise of single domains. Nat Biotechnol (2005) 23:1126–36. 10.1038/nbt1142 16151406

[61] Vanlandschoot P Stortelers C Beirnaert E Ibanez LI Schepens B Depla E Nanobodies^(R)^: new ammunition to battle viruses. Antiviral Res (2011) 92:389–407. 10.1016/j.antiviral.2011.09.002 21939690

[62] Unciti-Broceta JD Del Castillo T Soriano M Magez S Garcia-Salcedo JA. Novel therapy based on camelid nanobodies. Ther Deliv (2013) 4:1321–36. 10.4155/tde.13.87 24116915

[63] De Meyer T Muyldermans S Depicker A. Nanobody-based products as research and diagnostic tools. Trends Biotechnol (2014) 32:263–70. 10.1016/j.tibtech.2014.03.001 24698358

[64] Helma J Cardoso MC Muyldermans S Leonhardt H. Nanobodies and recombinant binders in cell biology. J Cell Biol (2015) 209:633–44. 10.1083/jcb.201409074 26056137 PMC4460151

[65] Bever CS Dong JX Vasylieva N Barnych B Cui Y Xu ZL V_H_H antibodies: emerging reagents for the analysis of environmental chemicals. Anal Bioanal Chem (2016) 408:5985–6002. 10.1007/s00216-016-9585-x 27209591 PMC4983233

[66] Li J Zhu Z. Research and development of next generation of antibody-based therapeutics. Acta Pharmacol Sin (2010) 31:1198–207. 10.1038/aps.2010.120 20694021 PMC4002304

[67] Mazor Y Sachsenmeier KF Yang C Hansen A Filderman J Mulgrew K Enhanced tumor-targeting selectivity by modulating bispecific antibody binding affinity and format valence. Sci Rep (2017) 7:40098. 10.1038/srep40098 28067257 PMC5220356

[68] Holliger P Winter G. Engineering bispecific antibodies. Curr Opin Biotechnol (1993) 4:446–9. 10.1016/0958-1669(93)90010-T 7763975

[69] Rozan C Cornillon A Petiard C Chartier M Behar G Boix C Single-domain antibody-based and linker-free bispecific antibodies targeting FcγRIII induce potent antitumor activity without recruiting regulatory T cells. Mol Cancer Ther (2013) 12:1481–91. 10.1158/1535-7163.MCT-12-1012 23757164

[70] Muruganandam A Tanha J Narang S Stanimirovic D. Selection of phage-displayed llama single-domain antibodies that transmigrate across human blood-brain barrier endothelium. FASEB J (2002) 16:240–2. 11772942 10.1096/fj.01-0343fje

[71] Abulrob A Sprong H Van Bergen en Henegouwen P Stanimirovic D. The blood-brain barrier transmigrating single domain antibody: mechanisms of transport and antigenic epitopes in human brain endothelial cells. J Neurochem (2005) 95:1201–14. 10.1111/j.1471-4159.2005.03463.x 16271053

[72] Li T Bourgeois JP Celli S Glacial F Le Sourd AM Mecheri S Cell-penetrating anti-GFAP V_H_H and corresponding fluorescent fusion protein V_H_H-GFP spontaneously cross the blood-brain barrier and specifically recognize astrocytes: application to brain imaging. FASEB J (2012) 26:3969–79. 10.1096/fj.11-201384 22730440

[73] Webster CI Caram-Salas N Haqqani AS Thom G Brown L Rennie K Brain penetration, target engagement, and disposition of the blood-brain barrier-crossing bispecific antibody antagonist of metabotropic glutamate receptor type 1. FASEB J (2016) 30:1927–40. 10.1096/fj.201500078 26839377

[74] Moutel S Bery N Bernard V Keller L Lemesre E de Marco A NaLi-H1: a universal synthetic library of humanized nanobodies providing highly functional antibodies and intrabodies. Elife (2016) 5:e16228. 10.7554/eLife.16228 27434673 PMC4985285

[75] Monegal A Ami D Martinelli C Huang H Aliprandi M Capasso P Immunological applications of single-domain llama recombinant antibodies isolated from a naive library. Protein Eng Des Sel (2009) 22:273–80. 10.1093/protein/gzp002 19196718

[76] Goldman ER Anderson GP Liu JL Delehanty JB Sherwood LJ Osborn LE Facile generation of heat-stable antiviral and antitoxin single domain antibodies from a semisynthetic llama library. Anal Chem (2006) 78:8245–55. 10.1021/ac0610053 17165813 PMC2528076

[77] Yan J Li G Hu Y Ou W Wan Y. Construction of a synthetic phage-displayed nanobody library with CDR3 regions randomized by trinucleotide cassettes for diagnostic applications. J Transl Med (2014) 12:343. 10.1186/s12967-014-0343-6 25496223 PMC4269866

[78] Ladenson RC Crimmins DL Landt Y Ladenson JH. Isolation and characterization of a thermally stable recombinant anti-caffeine heavy-chain antibody fragment. Anal Chem (2006) 78:4501–8. 10.1021/ac058044j 16808459

[79] Spinelli S Frenken LG Hermans P Verrips T Brown K Tegoni M Camelid heavy-chain variable domains provide efficient combining sites to haptens. Biochemistry (2000) 39:1217–22. 10.1021/bi991830w 10684599

[80] Smolarek D Hattab C Hassanzadeh-Ghassabeh G Cochet S Gutierrez C de Brevern AG A recombinant dromedary antibody fragment (V_H_H or nanobody) directed against human Duffy antigen receptor for chemokines. Cell Mol Life Sci (2010) 67:3371–87. 10.1007/s00018-010-0387-6 20458517 PMC2966875

[81] Traenkle B Emele F Anton R Poetz O Haeussler RS Maier J Monitoring interactions and dynamics of endogenous β-catenin with intracellular nanobodies in living cells. Mol Cell Proteomics (2015) 14:707–23. 10.1074/mcp.M114.044016 25595278 PMC4349989

[82] van der Linden RH Frenken LG de Geus B Harmsen MM Ruuls RC Stok W Comparison of physical chemical properties of llama V_H_H antibody fragments and mouse monoclonal antibodies. Biochim Biophys Acta (1999) 1431:37–46. 10.1016/S0167-4838(99)00030-8 10209277

[83] Doyle PJ Arbabi-Ghahroudi M Gaudette N Furzer G Savard ME Gleddie S Cloning, expression, and characterization of a single-domain antibody fragment with affinity for 15-acetyl-deoxynivalenol. Mol Immunol (2008) 45:3703–13. 10.1016/j.molimm.2008.06.005 18632156

[84] Maussang D Mujic-Delic A Descamps FJ Stortelers C Vanlandschoot P Stigter-van Walsum M Llama-derived single variable domains (nanobodies) directed against chemokine receptor CXCR7 reduce head and neck cancer cell growth in vivo. J Biol Chem (2013) 288:29562–72. 10.1074/jbc.M113.498436 23979133 PMC3795254

[85] McCoy LE Rutten L Frampton D Anderson I Granger L Bashford-Rogers R Molecular evolution of broadly neutralizing llama antibodies to the CD4-binding site of HIV-1. PLoS Pathog (2014) 10:e1004552. 10.1371/journal.ppat.1004552 25522326 PMC4270772

[86] Peyrassol X Laeremans T Gouwy M Lahura V Debulpaep M Van Damme J Development by genetic immunization of monovalent antibodies (nanobodies) behaving as antagonists of the human ChemR23 receptor. J Immunol (2016) 196:2893–901. 10.4049/jimmunol.1500888 26864035

[87] Liu S Wang S Lu S. DNA immunization as a technology platform for monoclonal antibody induction. Emerg Microbes Infect (2016) 5:e33. 10.1038/emi.2016.27 27048742 PMC4855071

[88] Nguyen VK Zou X Lauwereys M Brys L Bruggemann M Muyldermans S. Heavy-chain only antibodies derived from dromedary are secreted and displayed by mouse B cells. Immunology (2003) 109:93–101. 10.1046/j.1365-2567.2003.01633.x 12709022 PMC1782939

[89] Zou X Smith JA Nguyen VK Ren L Luyten K Muyldermans S Expression of a dromedary heavy chain-only antibody and B cell development in the mouse. J Immunol (2005) 175:3769–79. 10.4049/jimmunol.175.6.3769 16148123

[90] Janssens R Dekker S Hendriks RW Panayotou G van Remoortere A San JK Generation of heavy-chain-only antibodies in mice. Proc Natl Acad Sci U S A (2006) 103:15130–5. 10.1073/pnas.0601108103 17015837 PMC1586177

[91] Kohler G Milstein C Continuous cultures of fused cells secreting antibody of predefined specificity. Nature (1975) 256:495–7. 10.1038/256495a0 1172191

[92] Prentice HG Blacklock HA Janossy G Bradstock KF Skeggs D Goldstein G Use of anti-T-cell monoclonal antibody OKT3 to prevent acute graft-versus-host disease in allogeneic bone-marrow transplantation for acute leukaemia. Lancet (1982) 1:700–3. 10.1016/S0140-6736(82)92619-8 6122004

[93] Cosimi AB Colvin RB Burton RC Rubin RH Goldstein G Kung PC Use of monoclonal antibodies to T-cell subsets for immunologic monitoring and treatment in recipients of renal allografts. N Engl J Med (1981) 305:308–14. 10.1056/NEJM198108063050603 6454075

[94] Beck A Wurch T Bailly C Corvaia N. Strategies and challenges for the next generation of therapeutic antibodies. Nat Rev Immunol (2010) 10:345–52. 10.1038/nri2747 20414207

[95] Ecker DM Jones SD Levine HL. The therapeutic monoclonal antibody market. MAbs (2015) 7:9–14. 10.4161/19420862.2015.989042 25529996 PMC4622599

[96] Winter G Griffiths AD Hawkins RE Hoogenboom HR Making antibodies by phage display technology. Annu Rev Immunol (1994) 12:433–55. 10.1146/annurev.iy.12.040194.002245 8011287

[97] Hoogenboom HR. Selecting and screening recombinant antibody libraries. Nat Biotechnol (2005) 23:1105–16. 10.1038/nbt1126 16151404

[98] Nelson AL Reichert JM Development trends for therapeutic antibody fragments. Nat Biotechnol (2009) 27:331–7. 10.1038/nbt0409-331 19352366

[99] McCafferty J Griffiths AD Winter G Chiswell DJ. Phage antibodies: filamentous phage displaying antibody variable domains. Nature (1990) 348:552–4. 10.1038/348552a0 2247164

[100] Jespers LS Roberts A Mahler SM Winter G Hoogenboom HR. Guiding the selection of human antibodies from phage display repertoires to a single epitope of an antigen. Biotechnology (N Y) (1994) 12:899–903. 7521646 10.1038/nbt0994-899

[101] Kempeni J Preliminary results of early clinical trials with the fully human anti-TNF-α monoclonal antibody D2E7. Ann Rheum Dis (1999) 58(Suppl 1):I70–2. 10.1136/ard.58.2008.i70 10577977 PMC1766582

[102] Wang Y Fan Z Shao L Kong X Hou X Tian D Nanobody-derived nanobiotechnology tool kits for diverse biomedical and biotechnology applications. Int J Nanomedicine (2016) 11:3287–303. 10.2147/IJN.S107194 27499623 PMC4959585

